# Impact of Low Hemoglobin on Body Composition, Strength, and Redox Status of Older Hemodialysis Patients Following Resistance Training

**DOI:** 10.3389/fphys.2021.619054

**Published:** 2021-03-10

**Authors:** Victor da Silva, Hugo Corrêa, Rodrigo Neves, Lysleine Deus, Andrea Reis, Michel Souza, Cláudio dos Santos, Danilo de Castro, Fernando Honorato, Herbert Simões, Milton Moraes, Brad Schoenfeld, Jonato Prestes, Thiago Rosa

**Affiliations:** ^1^Graduate Program of Physical Education, Catholic University of Brasilia, Brasília, Brazil; ^2^Department of Nephrology, Federal University of São Paulo, São Paulo, Brazil; ^3^Federal University of Tocantins, Tocantins, Brazil; ^4^Department of Health Sciences, University of New York City, New York City, NY, United States

**Keywords:** exercise-training, oxidative stress, muscle mass, anemia, end-stage renal disease

## Abstract

**Introduction:**

The purpose of this study was to: (i) investigate the effect of six months of resistance training (RT) on body composition, muscle strength, hematological patterns, and redox profile in maintenance hemodialysis (HD) patients, and; (ii) evaluate the effects of baseline concentrations of hemoglobin on the RT response.

**Methods:**

One hundred fifty-seven subjects with chronic kidney disease (CKD) were randomly allocated into two groups: Control [CTL, (*n* = 76)] and RT (*n* = 81). A first visit was required for anamnesis and anthropometric measurements. Venous blood samples were collected at baseline and after twenty-four weeks of training in all patients for the analysis of clinical and redox balance markers. The RT program spanned six months and consisted of three sets of 8–12 repetitions with a rating of perceived exertion between 5 and 8 for three weekly sessions. Each exercise session was performed in twelve resistance exercises and it least for approximately 40 min.

**Results:**

The main results demonstrated that RT decreased waist circumference by 3%, and decreased thiobarbituric reactive species (TBARS) by 28%. Moreover, RT increased handgrip strength by 28.4%, fat-free mass by 4.1%, hemoglobin by 5%, iron by 33.4%, glutathione by 121%, and Trolox equivalent antioxidant capacity by 14.2% (*p* < 0.05). Low hemoglobin concentrations impaired the effect of RT on fat-free mass gain.

**Conclusion:**

Six months of RT in HD patients improved clinical parameters, such as hemoglobin, iron, body composition, and redox balance, while low hemoglobin concentration impaired exercise-benefits on fat-free mass in patients with CKD. These findings can contribute to a better clinical application of RT in the maintenance of hemodialysis patients.

## Introduction

Maintenance hemodialysis patients usually present muscle wasting and a high percentage of body-fat (Cheung et al., 2010), which decreases physical functionality and increases mortality risk (Sietsema et al., 2004). The combination of increased body fat and muscle wasting aggravates chronic kidney disease (CKD) and negatively affects the health status of the patient (Jhee et al., 2020). Furthermore, these patients often develop anemia due to the lower production of erythropoietin to stabilize the production of hemoglobin, and maintenance of iron homeostasis (Astor et al., 2002; Collister et al., 2017; Gafter-Gvili et al., 2019; Wong et al., 2019). The lack of treatment for these conditions increases oxidative stress, worsening the cardio-renal axis (Kopple et al., 2001; Mcclellan et al., 2004).

Furthermore, the oxidative damage strongly stimulates eryptosis which, in turn, increases the risk to the development of anemia (Bissinger et al., 2019). Taken together, this condition might negatively affect the microcirculation leading to undesirable comorbidities as cardiovascular diseases. In this regard, treatments that improve the redox status, strength and body composition may be an important tool in the prevention and rehabilitation of anemia in CKD patients.

Thus, the development of alternative therapies to improve body composition and oxidative stress may be an important intervention with high clinical utility for CKD patients. In this context, resistance training (RT) is an exercise modality that improves fat-free mass, strength, and physical functioning in frail, older individuals, as well as those with chronic diseases (Rosa et al., 2018). However, there is a divergence in the literature concerning the effect of RT on body composition and strength improvement in this population (Rosa et al., 2018), as some studies report an increase of skeletal muscle mass (Chen et al., 2010; Song and Sohng, 2012), while others do not (Cheema et al., 2007; Kopple et al., 2007; Marinho et al., 2016).

Rosa et al. (2018) speculated that these conflicting findings may be due to the lack of progressive overload during training, and thus sought to investigate whether progressive intradialytic RT could improve body composition, physical function, and quality of life in patients undergoing regular hemodialysis. Although the study demonstrated an improvement in muscle mass, strength, and bone mineral content, there was no effect on functional capacity, handgrip strength, or self-reported quality of life in these patients after the twelve-week intervention. Nevertheless, when observing the hemoglobin levels of participants in the control and intervention groups (11.50 ± 1.32 g/dL and 11.38 ± 1.17 g/dL, respectively), some patients displayed levels < 11 g/dL, which is not recommended for this population. Stray-Gundersen et al. (2016) demonstrated that the treatment for anemia and intradialytic exercise-training produced significant improvements in peak oxygen uptake and power in CKD patients. Therefore, a possible alternative explanation for the divergent responses to RT found previously in those with CKD is the confounding issue of low hemoglobin concentration. Therefore, it is already known that RT might act as an alternative therapy counteracting the CKD-related comorbidities (Corrêa et al., 2020; Moura et al., 2020; Neves et al., 2020). Nonetheless, it remains unknown if anemia or low hemoglobin impairs the aforementioned benefits on strength, body composition, and redox balance in hemodialysis patients.

To provide clarity on the topic, we aimed to: (i) investigate the effect of RT on body composition, muscle strength, hematological patterns, and redox profile in maintenance hemodialysis patients, and; (ii) evaluate the impact of baseline concentrations of hemoglobin on RT response. We hypothesized that RT would improve body composition, muscle strength, hematological patterns, and redox profile in this population. We also hypothesized that baseline concentrations of hemoglobin would at least partly explain the divergent responses to RT observed in previous research.

## Materials and Methods

### Subjects

At the beginning of the study, 202 patients volunteered for this study and were randomized by simple randomization (Suresh, 2011) in control (CTL) and resistance training (RT) group (101 patients each). In RT, 20 patients were lost to follow-up due to family issues, physical complications, and withdrawal. In CTL, 25 patients were lost to follow-up due to sleep complications, family issues, and withdrawal. Therefore, 76 patients were analyzed for CTL group and 81 for RT group (total *n* = 157). All one hundred and fifty-seven patients undergoing maintenance phase hemodialysis were analyzed for this research investigation (age: 67 ± 4 years; body mass: 73.4 ± 16 kg; body mass index: 27.05 ± 16 kg/m^2^). All experimental protocols were approved by the local University Ethic Committee, under the number: 23007319.0.0000.0029. Written informed consent was obtained from all the participants involved in the study. Inclusion criteria for participants were: (i) age ≥ 50; (ii) hemodialysis for at least three months; (iii) dialysis at least three times per week; and (iv) no significant medical complications in the last three months, except for vascular access correction. Exclusion criteria were as follows: (i) recent acute myocardial infarction within three months or unstable angina; (ii) systemic lupus erythematosus; (iii) congenital kidney malformation or some autoimmune disease that affects the kidneys; (iv) osteoarticular complications that could compromise physical exercise; v) decompensated heart failure that could limit participation in training; vi) severe decompensated diabetes; and vii) severe neuropathy, retinopathy, or diabetic nephropathy. Only patients who read, agreed, and signed the written informed consent participated in this study. All participants underwent nutritional counseling with a clinical nutritionist, while the CTL group did not receive any exercise intervention. All patients received the same guidelines from a multidisciplinary team: nutritionist, psychologist, social worker, nurse, pharmacist, and nephrologist.

At study’s end, we stratified the sample into groups with hemoglobin below 11g/dL (Hb < 11; CTL *n* = 20 and RT = 24) and hemoglobin above 11g/dL (Hb > 11g; CTL *n* = 56 and RT = 57) as per the guidelines of the American Society of Nephrology (Kalantar-Zadeh and Aronoff, 2009). All exercise testing and prescription procedures were carried out following the guidelines of the American College of Sports Medicine (Pescatello et al., 2014). Moreover, the present study was conducted in accordance with the guidelines and regulations for exercise prescription for end-stage renal disease (Segura-Orti and Johansen, 2010).

### Resistance-Training Protocol

The RT program was performed three times per week for 24 weeks and each training session took approximately 40 min. Participants performed three sets of 8–12 repetitions with the training load monitored using the OMNI rating of perceived exertion (RPE) scale (Robertson et al., 2003). The rest interval between sets and exercises was 120 s. Participants initially trained with a load corresponding to 8 repetitions at RPE of 5–6 for the first 12 weeks and 7–8 over the final 12 weeks. When the RPE indicated the load was too easy, we first increased the number of repetitions and, if the participant exceeded 12 repetitions, the load was increased.

The participants performed the pre-dialysis exercise approximately one hour before the start of their hemodialysis session. Each training session consisted of 12 exercises that included: chest press, squat, unilateral row, unilateral knee extension, unilateral knee flexion, unilateral shoulder press, hip thrust, unilateral biceps curl, unilateral hip adduction, unilateral elbow extension with dumbells, and seated calf raise. The e-Lastic (e-Sports Solutions, Brazil) cable was used to perform the chest press, unilateral row, and unilateral hip adduction exercises. For exercises performed with rubber bands, each repetition was counted by the application coupled to the e-Lastic dynamometer, allowing the measurement of the load used in each repetition. Dumbbells were used for performance of the unilateral shoulder press, unilateral biceps curl, unilateral elbow extension. Weighted cuffs were used for the unilateral knee extension (wrapped at the ankle), unilateral knee flexion (wrapped at the ankle), hip thrust (positioned at the hips), and seated calf raise (wrapped across the quadriceps). In the squat exercise, due to the fragility of the lower limbs of hemodialysis patients, we chose to use only body weight to perform the squat exercise with just four repetitions at the beginning of the protocol. For upper limb exercises, we prioritized unilateral exercises as a conservative measure to preserve arteriovenous fistula. The RT routine consisted of three sets of 8–12 repetitions with two minutes of rest between sets at a cadence of two seconds for both concentric and eccentric phases. All RT sessions were individually supervised by a strength and conditioning professional.

### Body Composition

All subjects were weighed on a mechanical scale (Filizola^®^, São Paulo, Brazil), and height was measured with a stadiometer built into the scale (precision: 0.5cm). Waist circumference was assessed at the level of umbilicus using an anthropometric tape (Sanny^®^, São Paulo, Brazil). Body fat and fat-free mass were measured using a Prodigy Advance Plus (LUNAR,Corp/General Electric; Madison, WI, United States) dual-energy X-ray absorptiometry (DXA) unit. Volunteers were asked to remove any metallic items they were wearing, such as rings, jewelry, belts, and watches (given that such objects affect the values of the estimated variables). Next, volunteers were placed in horizontal decubitus dorsal on the DXA table for full-body analysis. Before each use, the DXA equipment was calibrated according to the manufacturer’s recommendations, and cut line adjustments were predefined. All analyses were performed by the same evaluator.

### Handgrip Strength

Handgrip strength was measured with a hydraulic hand dynamometer (Jamar^®^ – Sammons Preston, Bolingbrook, United States), according to the American Society of Hand Therapists’ recommendations (Mathiowetz et al., 1984). Measurements were performed with participants in sitting position, elbow joint at 90°, forearm in a neutral position, and wrist between 0° and 30° of extension. The average of three attempts in the contralateral arm of the arteriovenous fistula, with 60 seconds rest between attempts, was registered in kgf.

### Biochemical Analysis

For lipid peroxidation, serum samples (80 μL) were diluted in 320 μL MiliQ H2O (1:5), and then 1 mL of 5 trichloroacetic acid (TCA) 17.5%, pH 2.0, and 1mL of thiobarbituric acid 0.6%, pH 2.0, were added, respectively. After homogenization, the samples were kept in a water bath for 30 min at 95°C. The reaction was interrupted with the immersion of the microtubes in ice, the addition of 1 mL of TCA 70%, pH 2.0, and incubation for 20 min at room temperature. After centrifugation (3,000 rpm for 15 min), the supernatant was removed and put in new microtubes and read by spectrophotometry at 540 nm. The concentration of lipid peroxidation products was calculated using the molar extinction coefficient equivalent for malondialdehyde (MDA-equivalent = 1.56⋅ 105 M^–1^ cm ^–1^). The coefficients of variability were <6%. Total antioxidant capacity was measured with a Trolox-equivalent assay kit (Quanti Chrom^®^ Bio Assay Systems, CA, United States). Reduced form of glutathione (GSH) was measured from plasma using an assay kit (Sigma Aldrich^®^, CA, United States) with a final spectrophotometric reading at 490 nm. Briefly, in a 96-well microplate, 20 μL of sample and 1,000 μL of working reagent were added to each well. After homogenizing and incubating at 37°C for 5 min, the absorbance was determined at 505 nm in a microplate reader (ELx800, Bio Tek, United States). For all antioxidant parameters, the intra-assay coefficient of variation was <4%. Hematological profile was assessed by the automated ABX Micros 60 analyzer in a private and standard commercial laboratory.

### Subgroup Stratification

Based on inferences from previous studies demonstrating anemia as a condition that influences peak oxygen uptake in CKD patients (Stray-Gundersen et al., 2016), we stratified the subgroups according to their serum hemoglobin levels (Hb < 11 or Hb > 11). Therefore, control group (CTL) was stratified in CTL Hb < 11 (*n* = 20) and CTL Hb > 11 (*n* = 56); and RT group was stratified in RT Hb < 11 (*n* = 23) and RT Hb > 11 (*n* = 58).

### Statistical Analysis

The primary endpoint of the present study was the effect of RT on strength and fat-free mass gain, and the secondary endpoint was the effect on redox balance. A third endpoint was to evaluate the effect of baseline hemoglobin levels on RT response. The normality and homogeneity of data were tested by the Kolmogorov-Smirnov and Levene tests, respectively. Data were expressed as medians (minimum – maximum) for baseline characteristics and means ± standard deviations to pre- and post-exercise training. The effect size was determined using Cohens’ *d.* Also, a 95% confidence interval (CI) was presented for the mean differences (Δ CTL – ΔRT) between groups. Given violations of normality, Chi-square and Mann-Whitney tests were used to analyze the baseline characteristics of individuals. Moreover, Mann-Whitney was also used to compare deltas (post – pre) of strength, fat-free mass gain and redox balance according to baseline Hb. Anthropometry, strength, clinical patterns (hemoglobin, iron, and ferritin) and redox balance were compared at pre- and post-training by the Kruskal-Wallis test followed by Dunns’ *post hoc*. Furthermore, Fishers exact test was performed to compare the proportions of the number of patients with Hb < 11 for both CTL and RT groups. Statistical significance was accepted at *p* < 0.05. Statistical analyses were performed using the GraphPadPrism6.0 (San Diego, United States).

## Results

One hundred fifty-seven patients completed the intervention. As presented in Table 1, there were no differences between groups for baseline characteristics. the loads observed at the beginning and at the end of RT program for e-Lastic cable, dumbbells, and weighted cuffs are presented in Table 2.

**TABLE 1:** Baseline characteristics.

Variables	CTL (*n* = 76)	RT (*n* = 81)	*p*-value
Age	66 (60–72)	68 (60–72)	NS
Sex, men/women	40♂/36♀	46♂/35♀	NS
Time of hemodialysis (months)	56 (35 – 70)	29.8 (15.5 – 51.1)	NS
Hypertension *n*, (%)	75 (100%)	81 (100%)	NS

*Data expressed as median (minimum and maximum values). CTL, control group; RT, resistance training; BMI, body mass index; NS, non-significant (*P* > 0.05). Chi-square was applied to compare the frequencies of distribution of men/women and patients with hypertension. Mann-Whitney test was applied to compare age, weight, BMI, waist circumference, fat-free mass and time of hemodialysis from baseline characteristics.*

**TABLE 2:** Loads pre- and post-intervention according to baseline status of Hb.

Exercises	RT Hb < 11 (*n* = 23)		RT Hb > 11 (*n* = 58)	
	Pre	Post	Pre	Post
e-Lastic exercises (kgf)	23.91 ± 7.99	63.74 ± 7.98†	23.63 ± 8.52	74.6 ± 11.57†
Dumbbells exercises (kg)	16.52 ± 6.73	34.22 ± 4.83†	15.19 ± 6.20	39.29 ± 6.19†
Weighted cuffs exercises (kg)	20.04 ± 6.44	63.35 ± 8.3†	18.4 ± 6.27	76.1 ± 12.61†

*Data expressed by mean ± standard deviation. RT, resistance training; Hb, hemoglobin. Kruskall-Wallis followed by Dunns *post hoc* tests were applied to compare groups. ^†^*p* < 0.0001 *vs* pre.*

The RT group displayed a greater improvement pre- to post *vs* CTL handgrip strength and TBARS (*p* < 0.0001). RT also showed greater pre- to post changes *vs* CTL in hemoglobin, iron and GSH (*p* < 0.0001). as displayed in Table 3.

**TABLE 3:** Pre, post, and delta within and between groups.

	CTL (*n* = 76)			RT (*n* = 81)			Delta CTL – Delta RT		
Variables	Pre	Post	Delta	Pre	Post	Delta	Estimate (95% CI)	ES	*P*-value
Body mass (kg)	72.7 ± 14.5	73.1 ± 15.4	0.42 ± 2.29	73.9 ± 16.6	71.7 ± 14.4	−2.2 ± 4.92	2.63(1.43−3.83)	0.68	0.874
Body mass index (kg/m^2^)	26.8 ± 2.9	26.9 ± 3.2	0.13 ± 0.81	27.2 ± 3.7	26.5 ± 3.2	−0.74 ± 1.77	0.88(0.45−1.31)	0.6	0.543
Waist circumference (cm)	95.4 ± 12.0	98.2 ± 12.0	2.71 ± 1.44	95.6 ± 11.7	92.7 ± 9.6	−2.92 ± 3.88	5.63(4.72−6.55)	1.9	0.034
Handgrip (kgf)	20.0 ± 5.1	19.7 ± 5.5	−0.34 ± 1.77	21.1 ± 4.3	27.1 ± 4.3^‡^	6 ± 2.8	−6.34(−7.07*to*−5.6)	0.9	<0.0001
Fat-free mass (kg)	42.6 ± 4.7	42.3 ± 5.1	−0.23 ± 1.46	43.0 ± 5.6	44.8 ± 6.5	1.78 ± 2.65	−2.01(−2.69*to*−1.34)	1.5	0.046
Body fat (kg)	30.1 ± 9.9	30.8 ± 10.5	0.66 ± 1.35	30.8 ± 11.0	26.8 ± 8.2	−3.99 ± 3.81	4.65(3.76−5.55)	2.7	0.079
Hemoglobin (g/dL)	11.7 ± 1.1	11.8 ± 1.2	0.04 ± 0.51	11.8 ± 1.3	12.4 ± 1.1	0.51 ± 0.61	−0.47(−0.65*to*−0.29)	0.9	0.032
Iron (mg/dL)	68.6 ± 14.4	67.6 ± 15.3	−1 ± 20.16	66.4 ± 16.1	88.6 ± 18.7^‡^,	22.22 ± 24.71	−23.22(−30.36*to*−16.08)	1.0	<0.0001
Ferritin (ng/mL)	401.7 ± 165.2	399.6 ± 165.0	−2.17 ± 11.74	456.1 ± 133.5	400.7 ± 126.5	−55.4 ± 53.18	53.23(41.18−65.27)	1.3	0.055
TBARS (mM)	13.2 ± 2.4	13.6 ± 2.6	0.39 ± 1.8	14.1 ± 2.3	11.0 ± 2.9^‡^,	−3.1 ± 1.8	3.51(2.92−4.09)	1.8	<0.0001
GSH (mM)	4.6 ± 1.9	5.0 ± 2.9	0.33 ± 2	4.2 ± 1.8	9.3 ± 2.0^‡^,	5.1 ± 1.1	−4.77(−5.3*to*−4.23)	2.9	<0.0001
TEAC (mM)	626.9 ± 191.5	589.5 ± 195.8	−37.4 ± 36.5	596.2 ± 218.2	680.7 ± 225.2	84.5 ± 62.7	−121.99(−138.08*to*−105.89)	2.3	0.014

*Data expressed by mean ± standard deviation. CTL: control group; RT: resistance training; CI: confidence interval; ES: effect size (cohens’ *d)* BMI: body mass index; TBARS: thiobarbituric acid reactive substances; GSH: glutathione; TEAC: Trolox equivalent antioxidant capacity. Kruskall-Wallis followed by Dunns *post hoc* tests were applied to compare CTL *vs* RT group.; **^‡^***p* < 0.0001 *vs.* CTL-post.*

**FIGURE 1:**
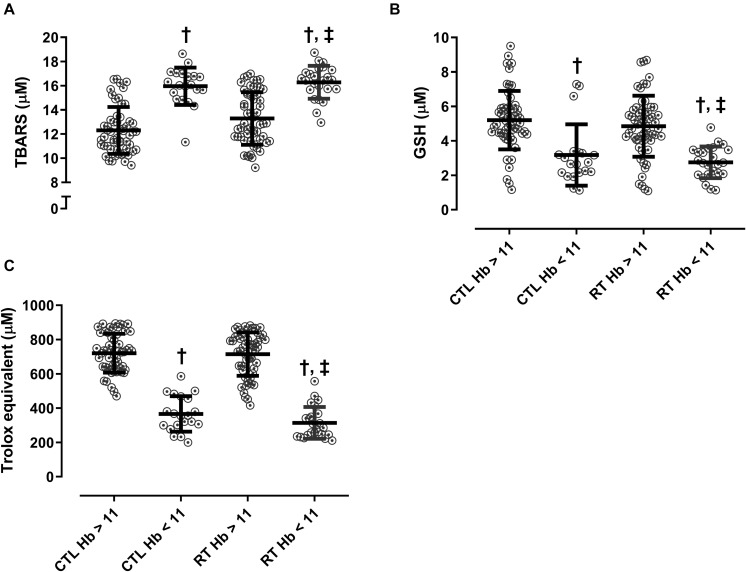
Baseline redox balance according to hemoglobin stratification, Hb < 11g/dl and Hb > 11g/dL. **(A)** TBARS, **(B)** GSH, and **(C)** trolox equivalent. TBARS: thiobarbituric acid reactive substances; GSH: glutathione; TEAC: Trolox equivalent antioxidant capacity; Hb: hemoglobin; CTL: control; RT: resistance training. Kruskal-Wallis test followed by Dunns’ *post hoc* were applied to compare groups. ^†^*p* < 0.05 *vs.* CTL Hb > 11, ^‡^*p* < 0.05 *vs.* RT Hb > 11.

Although RT promoted several improvements in patients with CKD, a considerable dispersion was noted in our data related to fat-free mass and redox balance. Considering that some patients started the protocol with a low concentration of hemoglobin (<11g/dL), which potentially could confound the data, we stratified the RT group according to baseline levels of hemoglobin to determine if lower levels of hemoglobin may have interfered with exercise-related benefits. Results showed that participants with lower hemoglobin levels at baseline presented lower markers of antioxidant defense and higher TBARS (*p* < 0.0001) as compared to participants with normal hemoglobin levels (Figure 1). It is worthy state that body composition and strength did not differ according to Hb levels at baseline. Moreover, the change percent for each group are presented in Table 4.

**TABLE 4:** Change percent for each group.

Variables	CTL Hb < 11 (%)	CTL Hb > 11 (%)	RT Hb < 11 (%)	RT Hb > 11 (%)
Body mass (kg)	+0.7	+0.5	–4.7	–2.3
Body mass index (kg/m^2^)	+0.6	+0.4	–4.5	–2.1
Waist circumference (cm)	+2.7	+2.8	–1	–3.8
Handgrip (kgf)	+1.5	–2.8	↑23.9	↑30.1
Fat-free mass (kg)	+0.1	–0.8	+ 1.2	+5.2
Body fat (kg)	+1.6	+1.4	↓13.7	↓12.6
Hemoglobin (g/dL)	–0.04	–0.4	↑11.8	+ 1.9
Iron (μg/dL)	+6.2	–3.9	↑51.5	↑26.6
Ferritin (ng/mL)	–1	–0.3	↓18.3	↓9.5
TBARS (μM)	+0.9	+4	↓21.2	↓22.4
GSH (μM)	+18.2	+4.8	↑189.3	↑104.5
TEAC (μM)	–4.6	–6	↑27.8	↑11.7

*CTL, control group; RT, resistance training; Hb, hemoglobin; BMI, body mass index; TBARS, thiobarbituric acid reactive substances; GSH, glutathione; TEAC, Trolox equivalent antioxidant capacity. + non-significant increase. – non-significant decrease. ↑ significant increase (*P* < 0.05). ↓ significant decrease (*P* < 0.05).*

**FIGURE 2:**
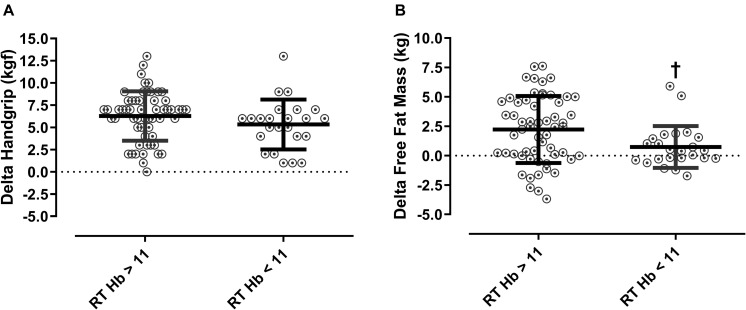
Delta Handgrip strength, and fat-free mass according to baseline hemoglobin concentrations in the RT group. Mann-Whitney test was applied to compare deltas. **(A)** Delta of handgrip strength and **(B)** delta fat-free mass. Hb: hemoglobin; RT: resistance training ^†^*p* < 0.0001 vs. RT Hb > 11.

As observed in Figure 2, patients from the RT group with low baseline levels of hemoglobin levels had an attenuated effect of RT on fat-free mass as compared with patients with normal hemoglobin (*p* < 0.0001).

**FIGURE 3:**
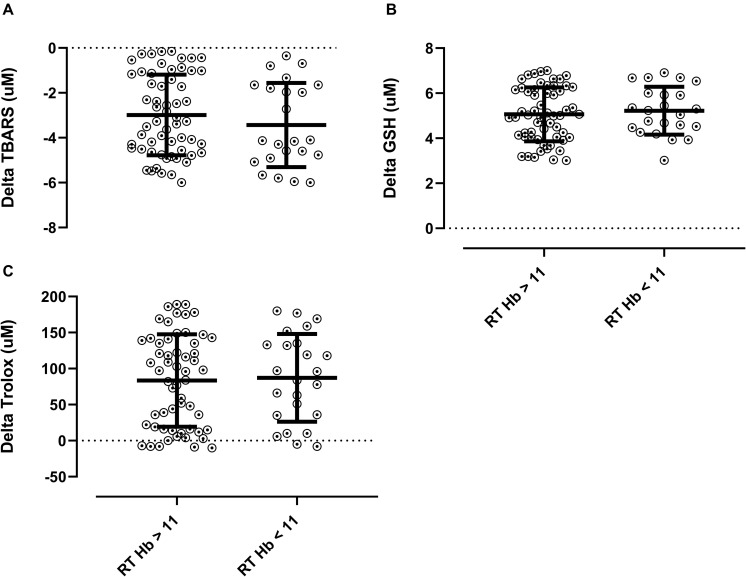
Delta Redox balance according to baseline concentration of hemoglobin. **(A)** Delta TBARS, **(B)** delta GSH, and **(C)** delta trolox. TBARS: thiobarbituric acid reactive substances; GSH: glutathione; TEAC: Trolox equivalent antioxidant capacity; Hb: hemoglobin; RT: resistance training. Mann-Whitney test was applied to compare groups.

There was no difference in TBARS, GSH, and Trolox when comparing low versus normal hemoglobin levels (*p* > 0.05). Described in Figure 3. The number of patients with Hb < 11 pre- and post- intervention are described on Table 5.

**TABLE 5:** Number of patients for each group pre- and post- intervention.

Conditions	Pre	Post	Total
CTL Hb < 10	20	19	39
CTL Hb > 10	56	57	113
RT Hb < 10	23	4	27
RT Hb > 10	58	77	135

*CTL, control group; RT, resistance training group; Hb, hemoglobin.*

## Discussion

We sought to determine the effect of RT on body composition, redox balance, and handgrip strength in maintenance hemodialysis patients. Despite the significant improvement in fat-free mass, strength and redox balance, our data showed a huge variation in response to the training protocol. Therefore, based on inferences from previous studies demonstrating anemia as a condition that influences peak oxygen uptake in CKD patients (Stray-Gundersen et al., 2016), we stratified the subgroups according to their blood hemoglobin levels. The main findings of the present study demonstrated that RT improves body composition, strength, and redox balance in CKD patients. Moreover, when stratified by hemoglobin levels, we observed that the effect of RT on fat-free mass gain is dependent on hemoglobin concentration in maintenance hemodialysis patients.

Anemia is extremely common among patients on hemodialysis, and can accentuate symptoms associated with decreased kidney function, such as fatigue, oxidative stress dyspnea and reduced exercise tolerance (Ye et al., 2018), which could possibly affect the development of strength and muscle hypertrophy. Although the Kidney Disease Improving Global Outcomes (KDIGO) guidelines have been used for anemia control goals (Ye et al., 2018), little is known about the impact of the patient’s initial hemoglobin level on the effects of physical training, especially for the objective of preventing sarcopenia and oxidative damage. Considering that sarcopenia and oxidative stress are constant issues in CKD, especially in maintenance hemodialysis patients (Johansen and Lee, 2015), the improvement of body composition and redox balance in the present study was an important finding for this population due to the strong risk for cachexia and frailty (Cheung et al., 2010; Von Haehling et al., 2017). As described by Johansen and Lee (2015), obesity is an increasing issue in end-stage renal patients; therefore, RT can be a potential interventional tool for the treatment/prevention of this condition, contributing to an enhanced body composition.

Hemodialysis patients have a strong susceptibility to acquire metabolic and nutrition complications which negatively affect handgrip strength (Garagarza et al., 2018). Garagarza et al. (2018) demonstrated that handgrip strength is highly correlated with lean tissue mass in hemodialysis patients. In this regard, a relevant finding of the present study is that RT promotes an increase in handgrip strength, which may prevent comorbidities related to muscle wasting (Von Haehling et al., 2017; Jhee et al., 2020). Nevertheless, when stratified by hemoglobin concentration, patients with anemia (<11g/dL in hemoglobin) did not realize RT-induced improvements in fat-free mass. This finding may be explained by evidence that lower levels of hemoglobin negatively affect the cardio-renal axis by increasing inflammation and oxidative stress (Collister et al., 2017; Nuhu and Bhandari, 2018; Gafter-Gvili et al., 2019), thus leading to muscle wasting (Jo et al., 2012) and, consequently, an increase in overall risk of cardiovascular outcomes.

Historically, was observed that low hemoglobin decreased the exercise performance of health subjects (Kaepovich and Millman, 1942; Balke et al., 1954; Calbet et al., 2006). Also, blood hemoglobin seems to decline according to the progression of CKD (Clyne et al., 1994). Therefore, low hemoglobin in hemodialysis patients appears to be a relevant factor to decrease exercise capacity in this population. This condition might be related to increased oxidative stress and low renal function in patients with Hb < 11 which significantly impairs the muscle mass gain (Moylan and Reid, 2007). However, further studies should evaluate the possible mechanisms related to the low hemoglobin impairing fat-free mass after RT. Another key finding of the present study is that just 4 patients continued with Hb < 11 after the intervention. Demonstrating that RT should be part of the treatment of anemia in this population. To the best of our knowledge, this is the first study that verified the influence of low hemoglobin on a long-term RT protocol, being clinically relevant in the prevention and treatment of CKD-related comorbidities.

A notable limitation of the present study is that we did not perform tissue biopsy analysis to investigate the iron balance before and after RT, while this data brings mechanistic insights of RT generating improvements in muscle mass, strength, body composition and redox balance in maintenance hemodialysis patients. However, our results demonstrated that muscle mass may depend on normal levels of hemoglobin (>11ng/L). In this regard, it seems that not only is aerobic training impaired by baseline hemoglobin levels (Stray-Gundersen et al., 2016), but resistance training as well, perhaps effectuated by different mechanisms. Furthermore, food intake, were not controlled in the present analysis. We recognize it as a potential limitation of the present study and encourage further studies to control this variable. Finally, we also encourage further studies to investigate the influence of the level of physical activity as a possible factor the influences RT response in this population.

The present study expands our knowledge of how RT affects body composition, handgrip strength and redox balance in maintenance hemodialysis patients. Given our findings, practitioners should seek to treat those with anemia or lower concentrations of hemoglobin before beginning a RT intervention in this population, as these conditions impair fat-free mass gain. Implementation of these findings can contribute to a better clinical application of RT in patients with CKD.

In conclusion, six months of RT improved clinical patterns, such as hemoglobin, iron, body composition and redox balance in HD patients. In addition, a low hemoglobin concentration impairs the exercise-benefits on fat-free mass. Nevertheless, RT should be recommended as a non-pharmacological interventional tool to improve the clinical status of this population.

## Data Availability Statement

The raw data supporting the conclusions of this article will be made available by the authors, without undue reservation.

## Ethics Statement

The studies involving human participants were reviewed and approved by Catholic University of Brasilia Ethics Committee: 23007319.0.0000.0029. The patients/participants provided their written informed consent to participate in this study.

## Author Contributions

VS, HC, and TR: conceptualization and project administration. VS, RN, LD, AR, CS, and TR: data curation. VS, HC, MS, LD, AR, and TR: formal analysis. VS, FH, HS, and MM: funding acquisition. VS, HC, DC, and TR: investigation. VS, DC, JP, and TR: methodology. VS, HC, BS, and TR: roles/writing – original draft. VS, HC, JP, BS, and TR: writing – review and editing. All authors contributed to the article and approved the submitted version.

## Conflict of Interest

The authors declare that the research was conducted in the absence of any commercial or financial relationships that could be construed as a potential conflict of interest.
